# Supplementary material for: In-plane topological p-n junction in the three-dimensional topological insulator Bi2−xSbxTe3−ySey
Source: Nat Commun. 2016 Dec 9;7:13763. doi: 10.1038/ncomms13763 (PMC5155151; doi:10.1038/ncomms13763)
Supplement: Supplementary Information — Supplementary Figures 1-6, Supplementary Notes 1-5 and Supplementary References [file ncomms13763-s1.pdf]

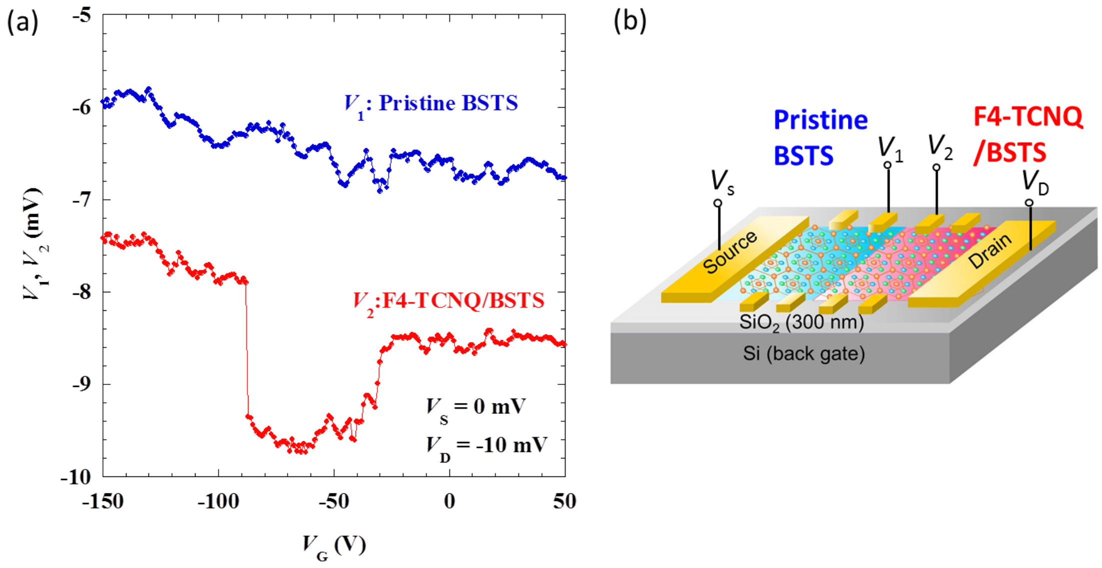

Supplementary Figure 1| Electrical transport across TPNJ. (a) Gate Voltage (**V**$_G$) dependence of **V**$_1$ (Pristine BSTS) and **V**$_2$ (F4-TCNQ/BSTS). (b) Schematic view of measurement setting in the present device. **V**$_G$ dependence of **V**$_1$ at the Pristine BSTS and **V**$_2$ at the F4-TCNQ/BSTS were measured using a source measurement unit of a semiconducting parameter analyzer.

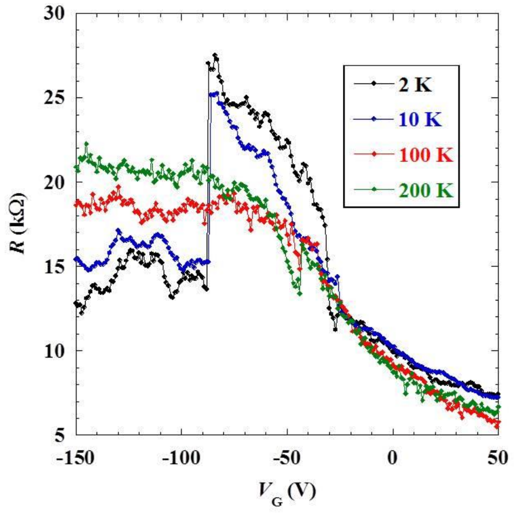

Supplementary Figure 2| Temperature dependence of electrical transport of TPNJ. Gate voltage (**V**$_G$) dependence of four probe resistance (R_{xx}) across the TPNJ made between pristine BSTS and F4-TCNQ/BSTS at various temperatures.

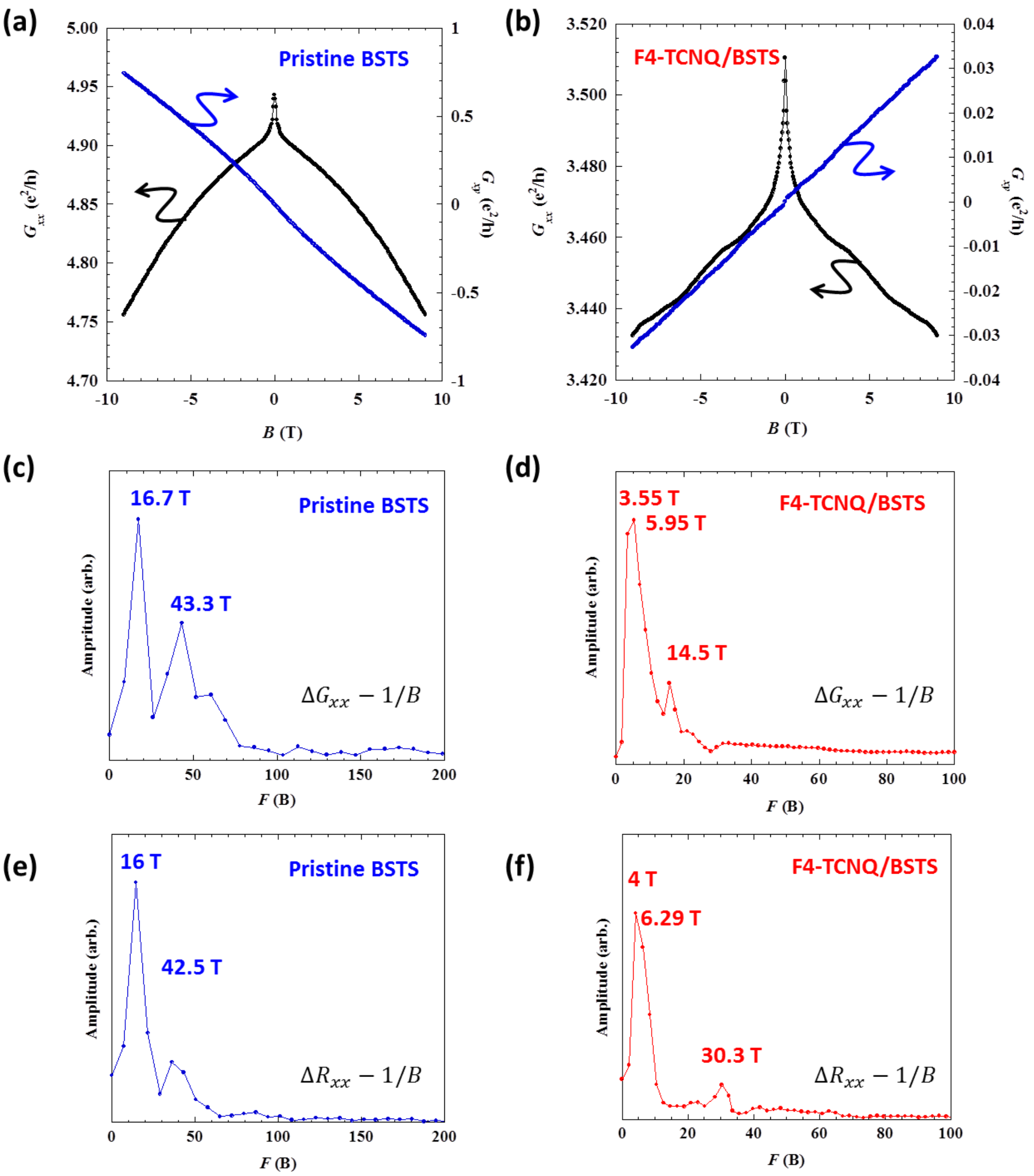

Supplementary Figure 3| Analyses of magnetotransport. Magnetic field (**B**) dependence of longitudinal Conductance G_{xx} and transverse Conductance G_{xy} for (a) pristine BSTS and (b) F4-TCNQ/BSTS. Results of fast Fourier transformation of (c), (d) $\Delta G_{xx} - 1/\mathbf{B}$ curves and (e), (f) $\Delta R_{xx} - 1/\mathbf{B}$ curves for pristine BSTS and BSTS/F4-TCNQ.

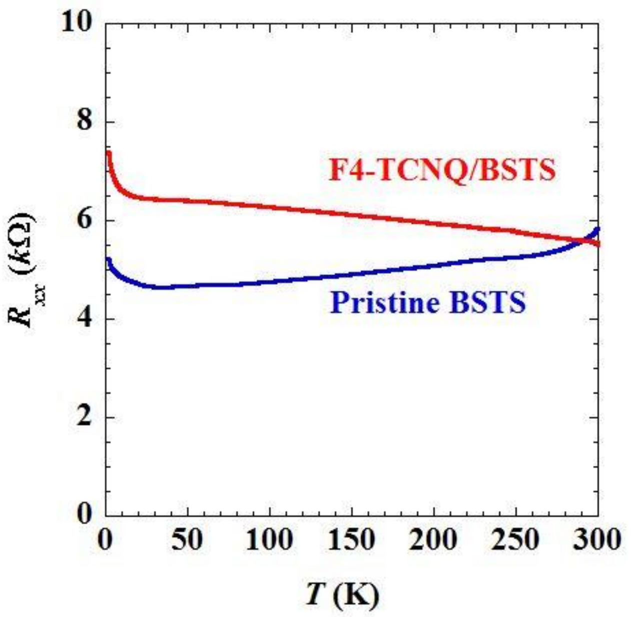

Supplementary Figure 4| Electrical transport of BSTS and F4-TCNQ/BSTS. Temperature dependence of electrical resistance for pristine BSTS and F4-TCNQ/BSTS. Metallic temperature dependence was observed in the pristine BSTS whereas it changed to a semiconducting-like one in F4-TCNQ/BSTS.

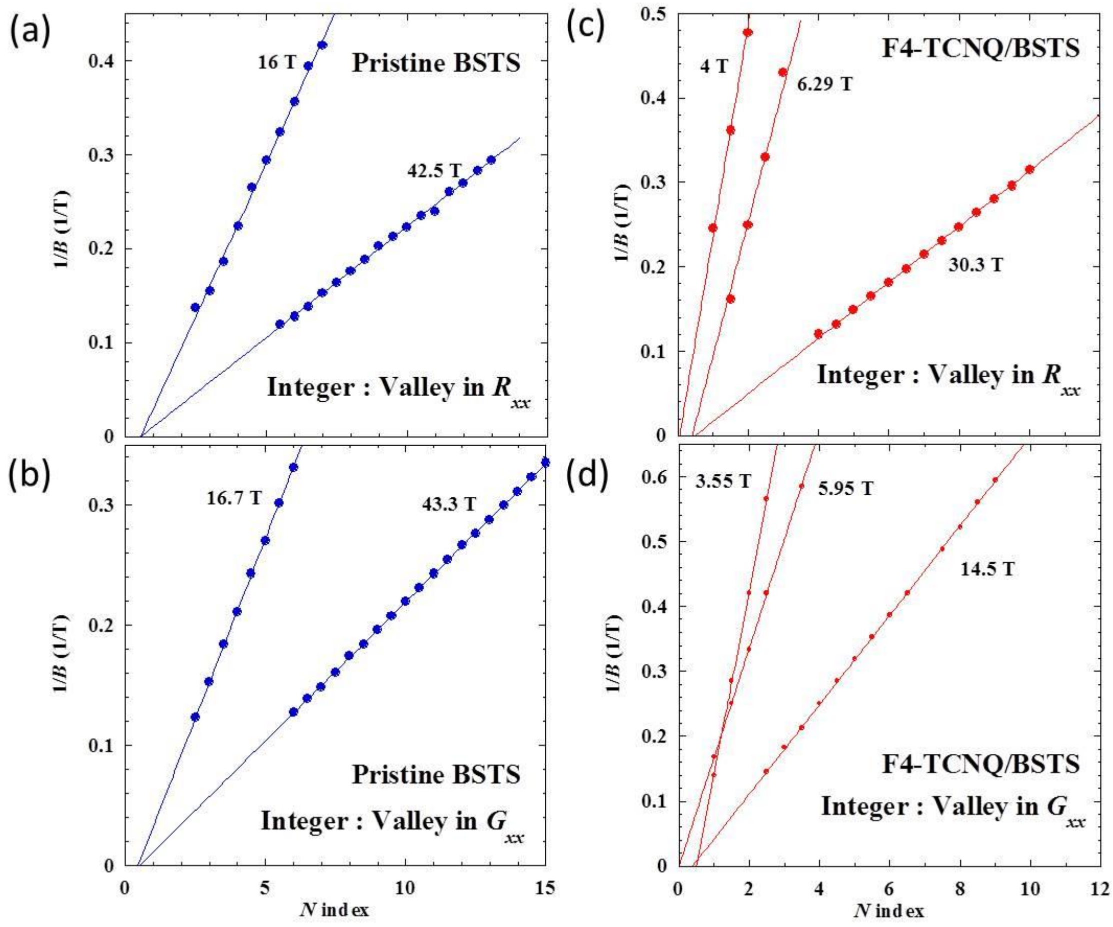

Supplementary Figure 5| Analyses of Landau level (LL) fan diagram. LL fan diagram plot of (a), (b) pristine BSTS (c), (d) F4-TCNQ/BSTS. These plots were made by employing the valleys and the peaks in the ΔR_{xx} and ΔG_{xx} based on the fast Fourier transformation of ΔR_{xx} − $1/\mathbf{B}$ and ΔG_{xx} − $1/\mathbf{B}$.

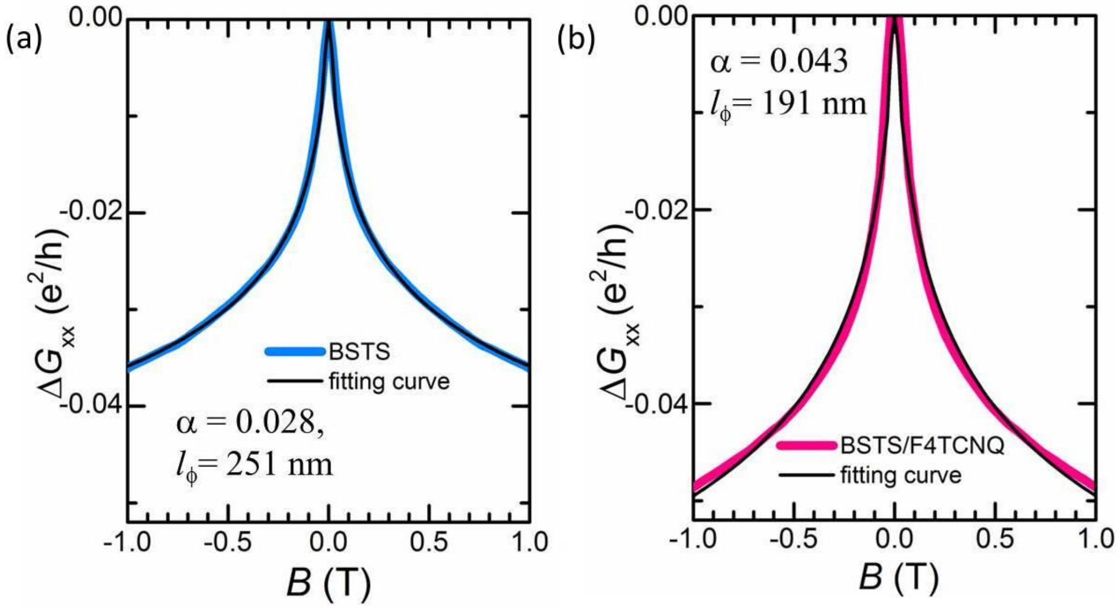

Supplementary Figure 6| Analyses of magnetoconductance curves for pristine BSTS and F4-TCNQ/BSTS. Black lines are the results of fitting using Hikami-Larkin-Nagaoka formula[4], $\delta G_{HLN} = \alpha \frac{e^2}{\pi h}\left[\Psi\left(\frac{\hbar}{4e\mathbf{B}l_{\emptyset}{}^2} + \frac{1}{2}\right) - \ln\left(\frac{\hbar}{4e\mathbf{B}l_{\emptyset}{}^2}\right)\right]$ where α and $l_{\emptyset}$ are the prefactor and the effective phase coherence length, respectively.

Supplementary Note 1 Gate voltage dependence of electric transport of topological p-n junction

Supplementary Fig. 4 (a) shows gate voltage ($\mathbf{V}_{\boldsymbol{G}}$) dependence of two different voltage channels using a source measurement unit of a semiconducting parameter analyzer. In the present measurement setting as shown in Supplementary Fig. 4 (b), a voltage drop (or jump) can be expected at the backward voltage electrode residing in the side of F4-TCNQ/BSTS when the source is in the side of pristine BSTS. For measurements, the source (the BSTS side) was fixed at 0 V and the drain (the F4-TCNQ/BSTS side) was set at the arbitrary values. A voltage change at the boundary between the pristine BSTS and the F4-TCNQ/BSTS region was detected by the $\mathbf{V}_{\boldsymbol{1}}$ (the BSTS side) and $\mathbf{V}_{\boldsymbol{2}}$ (the F4-TCNQ/BSTS side). When -10 mV was applied to the drain electrode, we clearly detected a voltage drop only at the backward voltage electrode of F4-TCNQ/BSTS ($\mathbf{V}_{\boldsymbol{2}}$) in the range of $\mathbf{V}_{\boldsymbol{G}}$ between -30 V and -90 V. This indicates that the resistance barriers against the flow of conduction electrons form as a consequence of the TPNJ or another accumulated band exactly located at the TPNJ between pristine BSTS and F4-TCNQ/BSTS.

Supplementary Note 2 Temperature dependence of electrical transport at the

topological p-n junction

Gate voltage (**V**$_G$) dependence of four-probe resistance (R_{xx}) across the TPNJ fabricated as the contact between pristine BSTS and F4-TCNQ/BSTS was shown at various temperatures (**T**) in Supplementary Fig. 5. An abrupt increase in R_{xx} was observed below 10 K, whereas it changes to a broad transition above 100 K. In the present TPNJ device, the high resistance TPNJ states can also switch off via an additional trivial surface accumulation layer on the bottom surface as discussed in the main text. The observed T dependent suppression of the high resistance TPNJ state can be understood in terms of both the reduction in the mean free pass in the topological Dirac-cone surface states[3] as well as the thermal excitation in the trivial surface accumulation layer with an increase in temperature.

Supplementary Note 3 Magnetoconductance analyzed using Hikami-Larkin-Nagaoka formula

Magnetoconductance of pristine BSTS and F4-TCNQ/BSTS was analyzed, using the Hikami-Larkin-Nagaoka (HLN) formula[4]: $\delta G_{HLN} = \alpha \frac{e^2}{\pi h}\left[\Psi\left(\frac{\hbar}{4e\mathbf{B}{l_\emptyset}^2} + \frac{1}{2}\right) - \right.$

$\ln(\frac{\hbar}{4e\mathbf{B}l_{\emptyset}^{2}})\Big]$ (Supplementary Eq. 1), where α and $l_{\emptyset}$ are the prefactor and the effective phase coherence length, respectively, as shown in supplementary Fig. 6. The analyses gave α = -0.028 and l_{ϕ} = 251 nm for pristine BSTS and α = -0.043 and l_{ϕ} = 191 nm for F4-TCNQ/BSTS. The smaller value in l_{ϕ} for F4-TCNQ/BSTS than that for pristine BSTS is indicative of increase in scattering by F4-TCNQ molecules on the top surface of BSTS thin films. The top and the bottom topological surface states can be confirmed in the SdH oscillations as described in the main text. The analysis of magnetoresistance using the HLN formula[5-7] generally gives α = -1 in the weak antilocalization limit, when the top and the bottom topological surface states are well separated by a good insulating bulk electronic states. It is noted that α = -0.5 is often observed when the insulation of the bulk band is not perfect[1]. Theoretical studies propose that α = 0.5 can be observed for the single channel in the weak localization limit when a trivial surface accumulation layer is present in the vicinity of bottom (top) and valence (conduction) bands in the 3D-TIs[8,9]. Since good insulating bulk electronic states as well as the top and bottom topological surface states were confirmed in the present BSTS films, compensation between the weak antilocalization on the topological surface states and the weak localization on the top and bottom surface accumulation layer can give a reasonable understanding for the small α value. Consequently, contribution from the additional

shallow surface accumulation layer originating from the band bending could be imagined from the viewpoint of small α values[8,9].

When we take a surface accumulation layer of the bulk into account as the origin of the rectification of spin-charged current, the following discussion can be made. In the conventional p-n diode mechanism in the framework of non-spin polarized bulk states, 3D-TI in the vicinity of the bottom of the conduction band (the top of the valence band) [10-12], a difference in resistance change can be expected between forward and backward bias voltage. However, no clear difference was observed between both directions and this is not the case. Moreover, the high resistance states in the TPNJ device cannot be simply understood in terms of the gate tuning of the trivial surface accumulation layers since the resistance in pristine BSTS and F4-TCNQ/BSTS is clearly lower than that of TPNJ device, indicating the scenario that the high resistance TPNJ switches on/off through the tuning of the trivial surface band by the negative $\mathbf{V}_{G}$ as discussed in the main text.

One may consider the Rashba splitting as a possible contribution[13]. However, the Rashba splitting would not be expected at the zero gate bias for both pristine BSTS and F4-TCNQ/BSTS based on the analyses of the magnetoconductance curves using HLN

formula and the fan diagram plot in the SdH oscillations. The effective contribution of the Rashba bands would not be large when the negative electric field is applied from the viewpoint of the band bending at the bottom surface. Our present experimental data contradict with the interpretation in terms of the Rashba bands on the bulk surface. Even if the Rashba band could be the case, it would also be questionable to consider a sharp resistance change, since Rashba splitting gradually develops with the strength of electric field.

Supplementary Note 4 Analysis of electrical transport of BSTS ultrathin film under magnetic field

As shown in the Fig. 3 (b) in the main text, both pristine BSTS and BSTS/F4-TCNQ showed large Shubnikov-de Haas (SdH) oscillations. By subtracting the background from the observed magnetoresistance (MR), the SdH oscillatory part was extracted. In 3D-TIs, the quantum oscillations are not generally apparent due to the influence of weak antilocalization on the surface states as well as the conventional MR in bulk, etc. In the present samples, however, we observed clear oscillations with sufficient amplitude and were able to distinguish the SdH oscillations. We employed both magnetoresistance and magnetoconductance curves by dividing them in the several

areas of **B** and then fitted them using a polynomial function: R_{xx} = a+ b***B** +c***B**^{2} and G_{xx} = a+ b***B** +c***B**^{2} in order to subtract the background as shown in Fig. 3 (c) and (d) of the main text. The oscillations of the SdH intensity as a function of 1/**B** were subjected to the fast Fourier transformation to determine the featured oscillation frequencies.

The resultant FFT curves evaluated from the G_{xx}-1/**B** curves and the R_{xx}-1/**B** curves are shown in the Spplementary Fig. 2 (c)-(f). Supplementary Fig. 3 (a) – (d), the Landau level fan diagram plots were made for pristine BSTS and F4-TCNQ/BSTS using the valley (the peak) in ΔR_{xx} or those in ΔG_{xx} as a function of integer n (a half integer n + 1/2). The analysis on R_{xx} gives 16 T and 42.5 T for pristine BSTS and 4 T, 6.29 T, and 30.3 T for F4-TCNQ/BSTS. The fan diagram plots provided the intercept values of 0.3508 (16 T) and 0.527 (42.5 T) for pristine BSTS and 0.054 (4 T), 0.410 (6.29 T), and 0.497 (30.3 T) for F4-TCNQ/BSTS. On the other hand, the analysis on G_{xx} gives 0.455 (16.7 T) and 0.505 (43.3 T) for pristine BSTS and 0.498 (3.55 T), 0.008 (5.95 T), and 0.390 (14.5 T) for F4-TCNQ/BSTS. The oscillation frequency and the intercept values in the fan diagram plots are consistent in the both analyses for pristine BSTS, but they are different in the case of F4-TCNQ/BSTS. In the 3D-TIs, because of the multi transport channels, analysis of ΔG_{xx}-1/**B** in the SdH oscillations usually is

reported gives more accurate results than that of ΔR_{xx}-1/$\mathbf{B}$ [2]. Here we employed the results from ΔG_{xx}-1/$\mathbf{B}$. In the present analyses, the two oscillations in each pristine BSTS and F4-TCNQ/BSTS were assigned to the nontrivial finite π Berry's phases corresponding to the top and the bottom topological surface states and one oscillation from F4-TCNQ/BSTS was ascribed to the trivial surface accumulated band.

Supplementary Note 5 Temperature dependence of electrical transport of $Bi_{2-x}Sb_xTe_{3-y}Se_y$ ultrathin film

Temperature dependence of electrical resistance (R_{xx}) for pristine $Bi_{2-x}Sb_xTe_{3-y}Se_y$ (BSTS) and F4-TCNQ/BSTS on a mica substrate was shown in Supplementary Fig. 1. Pristine BSTS showed a metallic temperature dependence in a wide range of temperature ($\mathbf{T}$), whereas F4-TCNQ/BSTS showed an insulting like behavior with higher resistance than that of pristine BSTS. This suggests that F4-TCNQ molecules increase scatterings on the surface electronic states in addition to the role as an electron acceptor. Note that the metallic behavior in pristine BSTS does not originate from the contribution of metallic bulk states. Thickness dependence of R_{xx} – $\mathbf{T}$ curves clearly showed a change from a semiconducting-like to a metallic-like electrical transport in a range of 10 – 50 nm in thickness, demonstrating that the contribution of the metallic

surface states can be tuned as a function of film thickness depending on the condition of insulating bulk electronic states. It should be noted that the observed resistance values of our samples were one order of magnitude higher than those of Bi_2Se_3 high quality thin films[1] and the metallic bulk band of the latter gives large contributions in electrical conductance[1] while it is negligibly small in our high quality thin films.

Supplementary References

[1] Taskin, A. A. Sasaki, S. Segawa, K. & Ando, Y. Manifestiation of topological protection in transport properties of epitaxial Bi_2Se_3 thin films, *Phys. Rev. Lett.* **109**, 066803 (2012).

[2] Ando, Y. Topological insulating materials, *J. Phys. Soc. Jpn.* **82**, 102001 (2013).

[3]Shiomi, Y. *et al.*, Spin-electricity conversion induced by spin injection into topological insulators. *Phys. Rev. Lett.* **113**, 196601 (2014).

[4] Hikami, S. Larkin, A. & Nagaoka, Y. Spin-Orbit Interaction and Magnetoresistance in the Two Dimensional Random System, *Prog. Theor. Phys.* **63**, 707-709 (1980).

[5] Ando, T. Nakanishi, T. & Saito, R. Berry's Phase and Absence of Back Scattering in Carbon Nanotubes, *J. Phys. Soc. Jpn.* **67**, 2857-2862 (1998).

[6] Fu, L. Kane, C. L. & Mele, E. J. Topological Insulators in Three Dimensions, *Phys. Rev. Lett.* **98**, 106803 (2007).

[7] Chen, J. *et al*., *Phys. Rev. Lett*. **105**, 176602 (2010).

[8] Lu, H. Z. & Shen, S. Q. Weak localization of bulk channels in topological insulator thin films, *Phys. Rev. B* **84**, 125138 (2011).

[9] Garate, I. & Glazman, L. Weak localization and antilozalization in topological insulator thin films with coherent bulk-surface coupling, *Phys. Rev. B* **86**, 035422 (2012).

[10] Shen, S. Q. Shan, W. Y. & Lu, H. Z. Topological insulator and the Dirac equation, *SPIN* **1**, 33 (2011).

[11] Zhang, H. J. Liu, C. X. Qi, X. L., Dai, X. Fang, & Z. Zhang, S. C. Topological insulators in Bi_2Se_3, Bi_2Te_3 and Sb_2Te_3 with a single Dirac cone on the surface *Nat. Phys.* **5**, 438-442 (2009).

[12] Liu, C. X. Qi. X. L. Zhang, H. J. Dai. X. Fang, Z. & Zhang, S. C., Model Hamiltonian for topological insulators, *Phys. Rev. B* **82**, 045122 (2010).

[13] Yang, F. *et al.*, Switching of charge-current-induced spin polarization in the topological insulator $BiSbTeSe_2$, *Phys. Rev. B* **94**, 075304 (2016).
